# Massive Pulmonary Embolism Presenting as Recurrent Cardiac Arrest: Successful Resuscitation and Thrombolysis in a 74-Year-Old Male

**DOI:** 10.7759/cureus.106909

**Published:** 2026-04-12

**Authors:** Pranali R Dave, Mohammad Z Khan, James R Higgins

**Affiliations:** 1 Medical School, California University of Science and Medicine, Colton, USA; 2 Cardiology, University of California, Los Angeles, Los Angeles, USA

**Keywords:** bedside echocardiography, cardiac arrest, computed tomography pulmonary angiography, deep vein thrombosis (dvt), massive pulmonary embolism, mcconnel sign, pulseless electric activity, right ventricular dysfunction, systemic thrombolysis, tenecteplase (tnk)

## Abstract

Massive pulmonary embolism (PE) is a life-threatening emergency often presenting with hemodynamic collapse or cardiac arrest. Despite advances in resuscitation and reperfusion therapies, outcomes remain poor. We report the case of a 74-year-old man with a history of hypertension, hyperlipidemia, and diabetes mellitus who presented with acute severe dyspnea leading to pulseless electrical activity (PEA) cardiac arrest. Return of spontaneous circulation was achieved with advanced cardiac life support, but he subsequently experienced recurrent PEA arrest requiring intubation, vasopressors, and antiarrhythmic therapy. Electrocardiography showed sinus tachycardia with right bundle branch block and repolarization abnormalities. Laboratory evaluation revealed marked troponin elevation, leukocytosis, thrombocytopenia, and hypokalemia. Duplex ultrasound demonstrated bilateral lower extremity deep vein thromboses. Echocardiography showed preserved left ventricular function with an enlarged, hypokinetic right ventricle and apical sparing. Chest computed tomography angiography revealed a large burden of bilateral pulmonary emboli with right ventricular strain and wedge-shaped pulmonary infarctions. The patient was treated with systemic thrombolysis (tenecteplase), complicated by transient oral and urinary bleeding that resolved spontaneously. Over several days, he stabilized, was weaned from vasopressors and mechanical ventilation, and discharged on full anticoagulation to outpatient follow-up. This case highlights the diagnostic challenge of massive PE, the value of bedside echocardiography, and the importance of prompt reperfusion therapy in unstable patients.

## Introduction

Pulmonary embolism (PE), a condition in which a blood clot blocks blood flow within the pulmonary arteries, is the third most common cause of cardiovascular mortality worldwide, following myocardial infarction and stroke [1,2]. With an annual incidence of approximately 60 to 120 cases per 100,000 population, PE affects nearly 10 million individuals globally each year and accounts for an estimated 60,000 to 100,000 deaths annually in the United States alone [2,3]. The burden of this disease is substantial, with total annual healthcare costs ranging from $7 to 10 billion in the United States [1,3].

Mortality is highest in cases of massive PE, defined by sustained hypotension, obstructive shock, or cardiac arrest. High-risk PE patients presenting with hemodynamic instability face in-hospital mortality rates of 20% to 42%, with the highest mortality, approaching 74%, observed in those who experience cardiac arrest [4,5]. Among patients who arrest from PE, only 16% survive to 30 days even with thrombolytic therapy, compared to 6% without it [6]. Current guidelines emphasize the importance of rapid diagnosis and early reperfusion therapy in these presentations, as systemic thrombolysis has been shown to reduce mortality and recurrence of PE compared to anticoagulation alone [1,7]. However, this benefit comes at the cost of increased major hemorrhage (6.3% vs. 1.5%) and a 2-5% risk of intracranial hemorrhage, which complicates clinical decision-making in unstable patients [2,8].

Despite advances in imaging, catheter-based interventions, and surgical thrombectomy, systemic thrombolysis remains the most widely available and rapidly deployable treatment for unstable patients with massive PE [9]. The educational value of this case lies in demonstrating the critical importance of maintaining clinical suspicion for PE in patients presenting with unexplained cardiac arrest, the utility of bedside echocardiography in identifying right ventricular dysfunction as a diagnostic clue, and the life-saving potential of prompt systemic thrombolysis despite bleeding risks. This case also illustrates successful management of recurrent pulseless electrical activity (PEA) arrest, a presentation associated with exceptionally high mortality, and highlights the feasibility of achieving favorable neurologic outcomes even after multiple resuscitations when reperfusion therapy is administered expeditiously.

We present a case of massive bilateral PE with recurrent cardiac arrest, managed successfully with advanced cardiac life support (ACLS), bedside imaging, and systemic thrombolysis, followed by stabilization and recovery.

## Case presentation

A 74-year-old man with a history of hypertension, hyperlipidemia, and diabetes mellitus presented with a sudden-onset severe dyspnea that awoke him from sleep. He called emergency medical services (EMS); on their arrival, he was in severe respiratory distress with cyanosis. En route to the hospital, he suffered a PEA cardiac arrest and underwent ACLS, with return of spontaneous circulation (ROSC) achieved before hospital arrival.

Upon arrival at the emergency department, his vital signs were a temperature of 96.6°F, heart rate of 133 beats/minute, blood pressure of 81/49 mmHg, and oxygen saturation of 75% on supplemental oxygen. Shortly thereafter, he again lost pulses, developing a recurrent PEA cardiac arrest. ACLS was resumed with administration of two doses of epinephrine, along with endotracheal intubation, and ROSC was subsequently achieved.

An electrocardiogram demonstrated sinus tachycardia with a right bundle branch block and secondary repolarization abnormalities, but no ST-segment elevations to suggest myocardial infarction. He was initially treated with aspirin and an intravenous heparin infusion while acute coronary syndrome remained in the differential diagnosis during early evaluation. A central venous catheter was placed, and vasopressor support with a phenylephrine infusion was initiated. Cardiology was consulted; however, emergent coronary angiography was not pursued, as it was thought unlikely to provide benefit.

Laboratory evaluation was notable for an elevated high-sensitivity troponin of 305 ng/L (reference <20 ng/L), rising to 1,265 ng/L one hour later, creatinine of 1.3 mg/dL, potassium of 2.9 mmol/L, white blood cell count of 11.4 × 10⁹/L, and platelet count of 136 × 10⁹/L. Electrolyte abnormalities were closely monitored, and potassium was repleted during resuscitative management, given the increased risk of arrhythmias. Duplex ultrasonography of the lower extremities revealed deep vein thromboses in the right peroneal and left popliteal veins.

**Figure 1:**
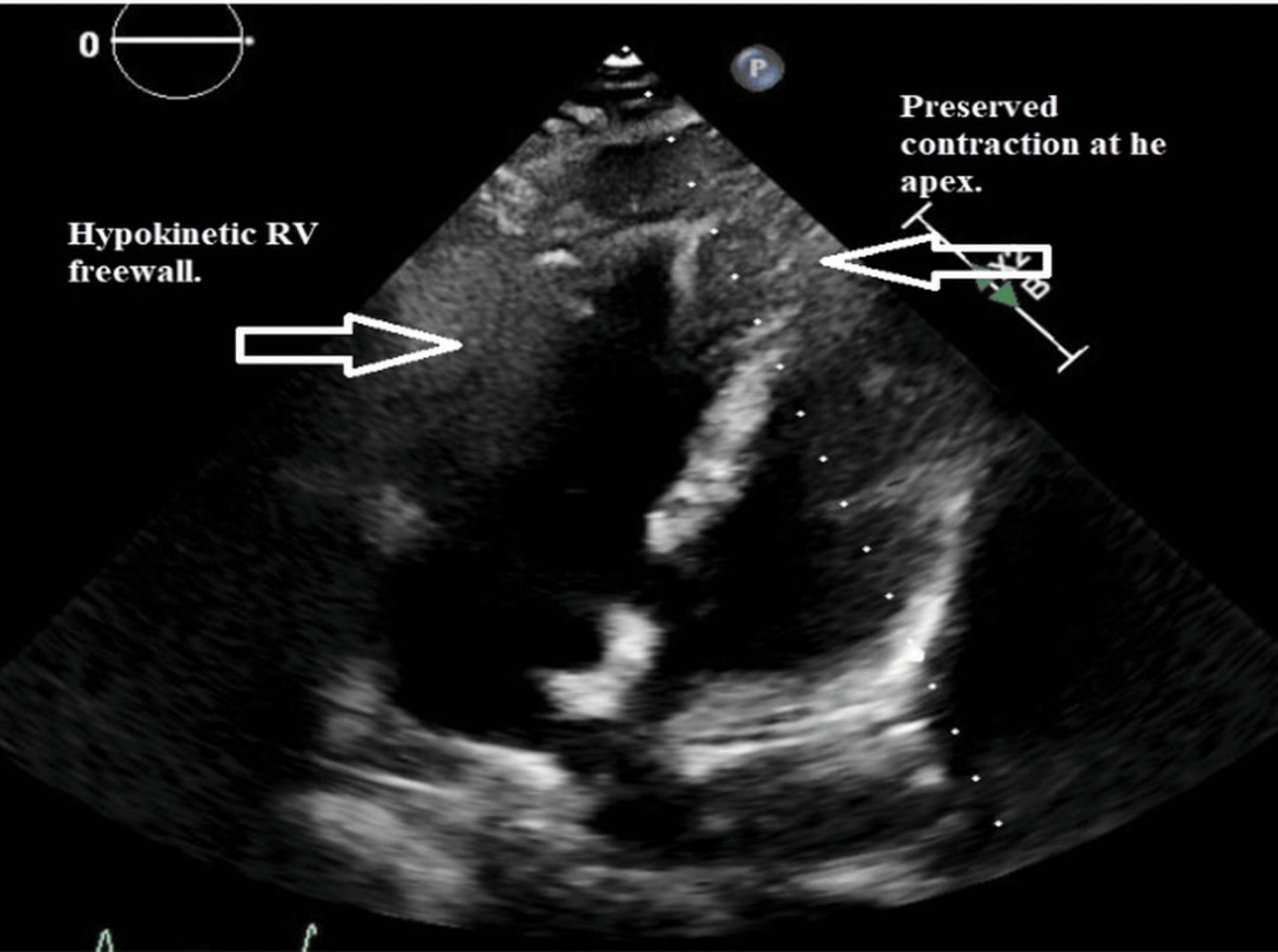
Apical four-chamber transthoracic echocardiogram demonstrating a severely dilated right ventricle (RV) with preserved apical contractility (McConnell’s sign; arrows).

**Figure 2:**
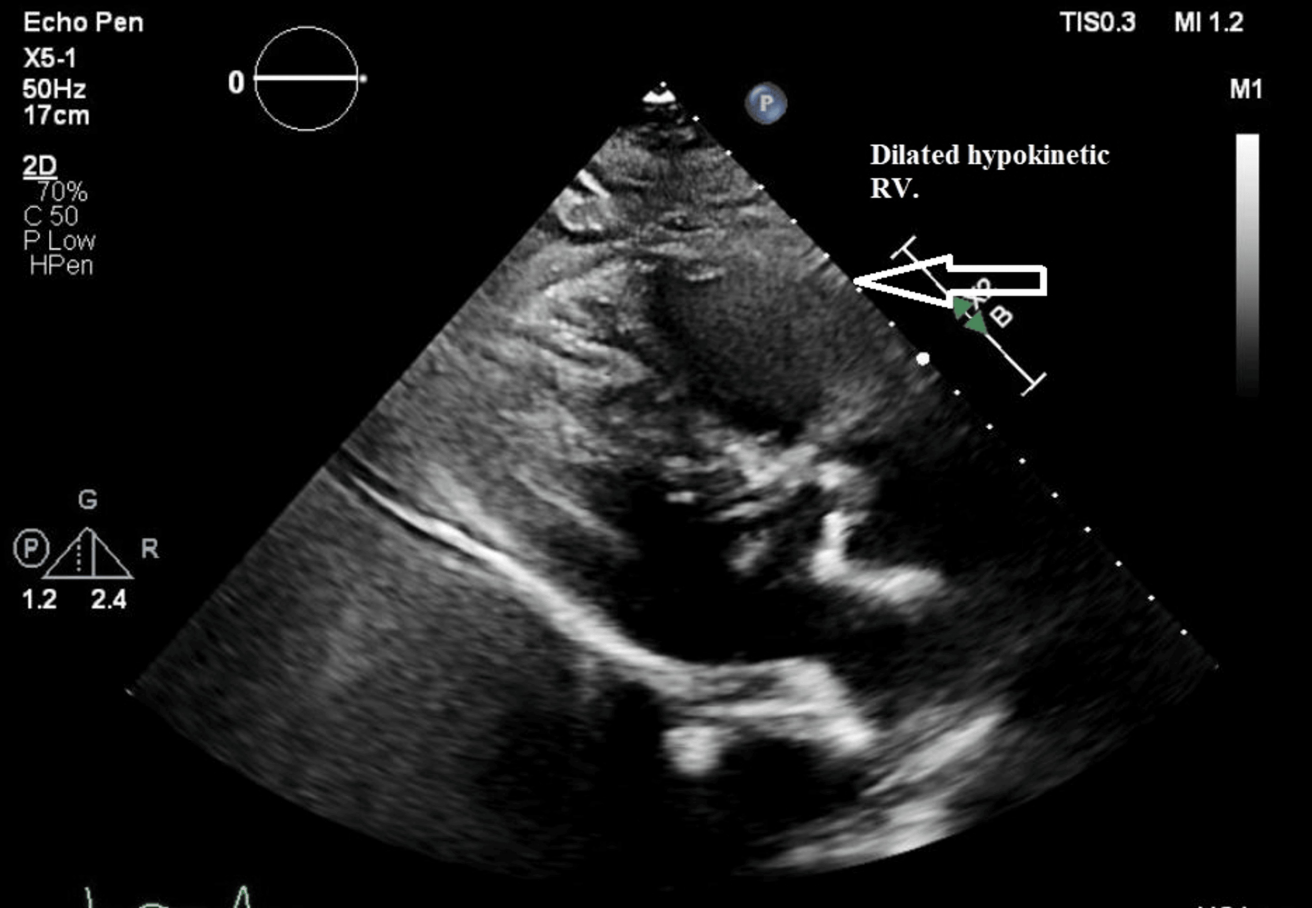
Parasternal short-axis echocardiographic view demonstrating flattening of the interventricular septum (arrow), resulting in a “D-shaped” left ventricle consistent with acute right ventricular pressure overload.

**Figure 3:**
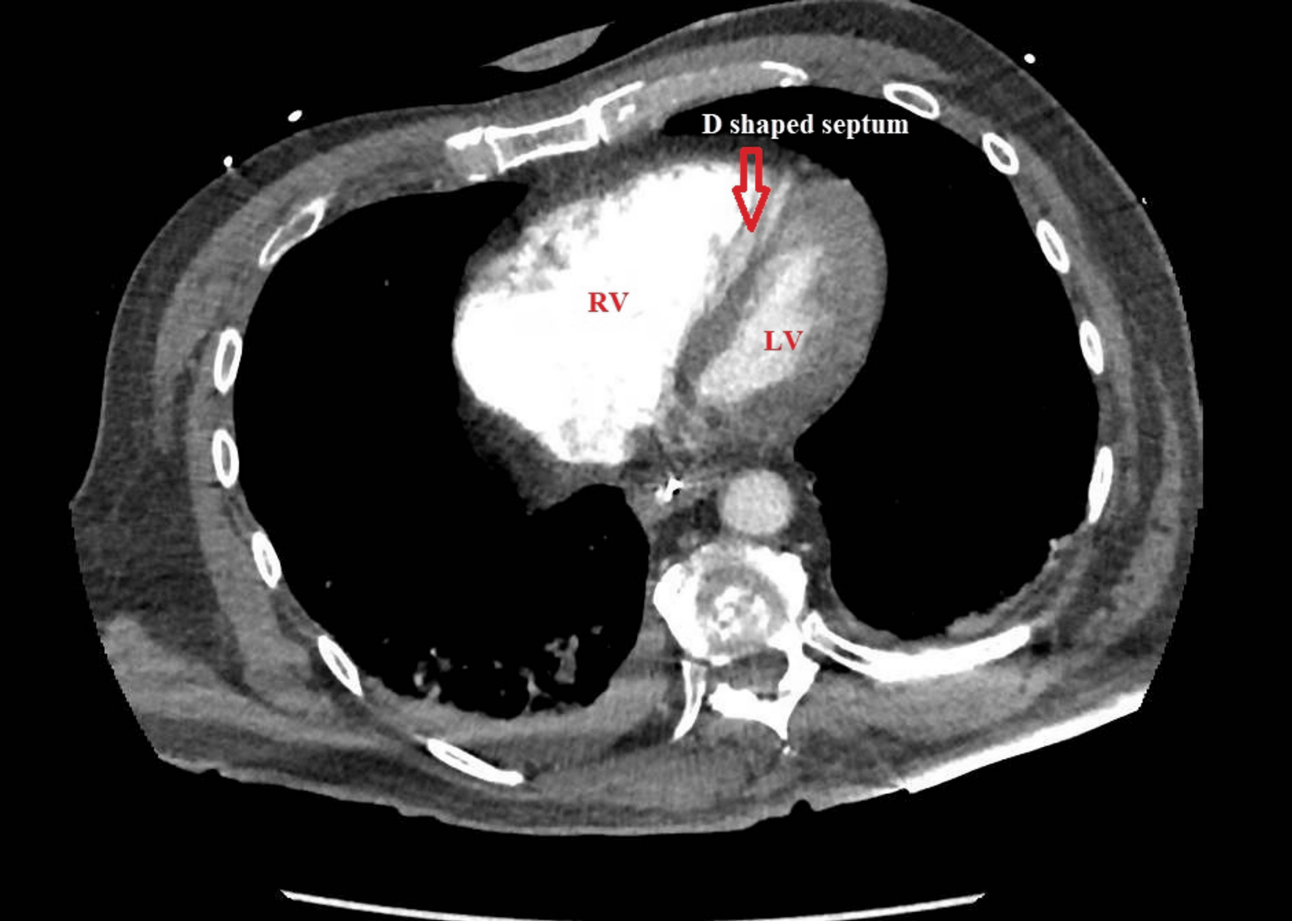
Axial chest computed tomography angiography image demonstrating flattening of the interventricular septum (arrow), resulting in a “D-shaped” left ventricle consistent with acute right ventricular pressure overload.

Transthoracic echocardiography showed preserved left ventricular systolic function but a severely dilated, hypokinetic right ventricle with apical sparing, consistent with McConnell’s sign [10]. In addition, the interventricular septum was flattened on the short-axis view, resulting in a “D-shaped” left ventricle, indicative of acute right ventricular pressure overload (Figures 1-3).

**Figure 4:**
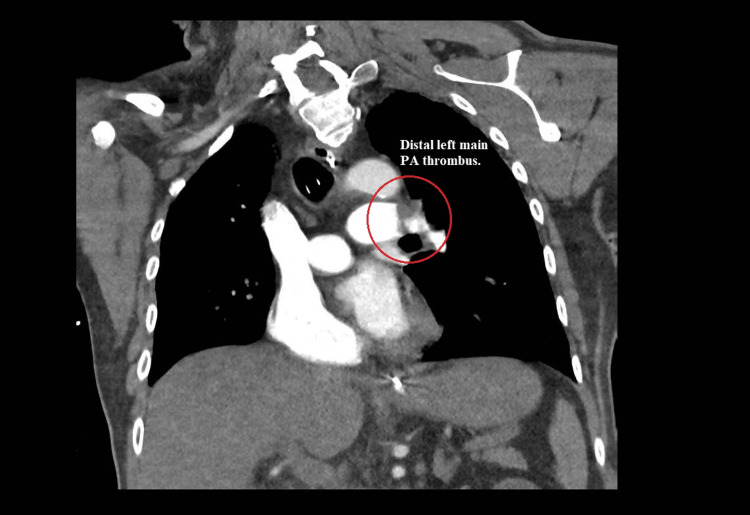
Coronal chest computed tomography angiography (lung window) demonstrating a filling defect in the distal left main pulmonary artery (circled region), consistent with an acute pulmonary embolus.

**Figure 5:**
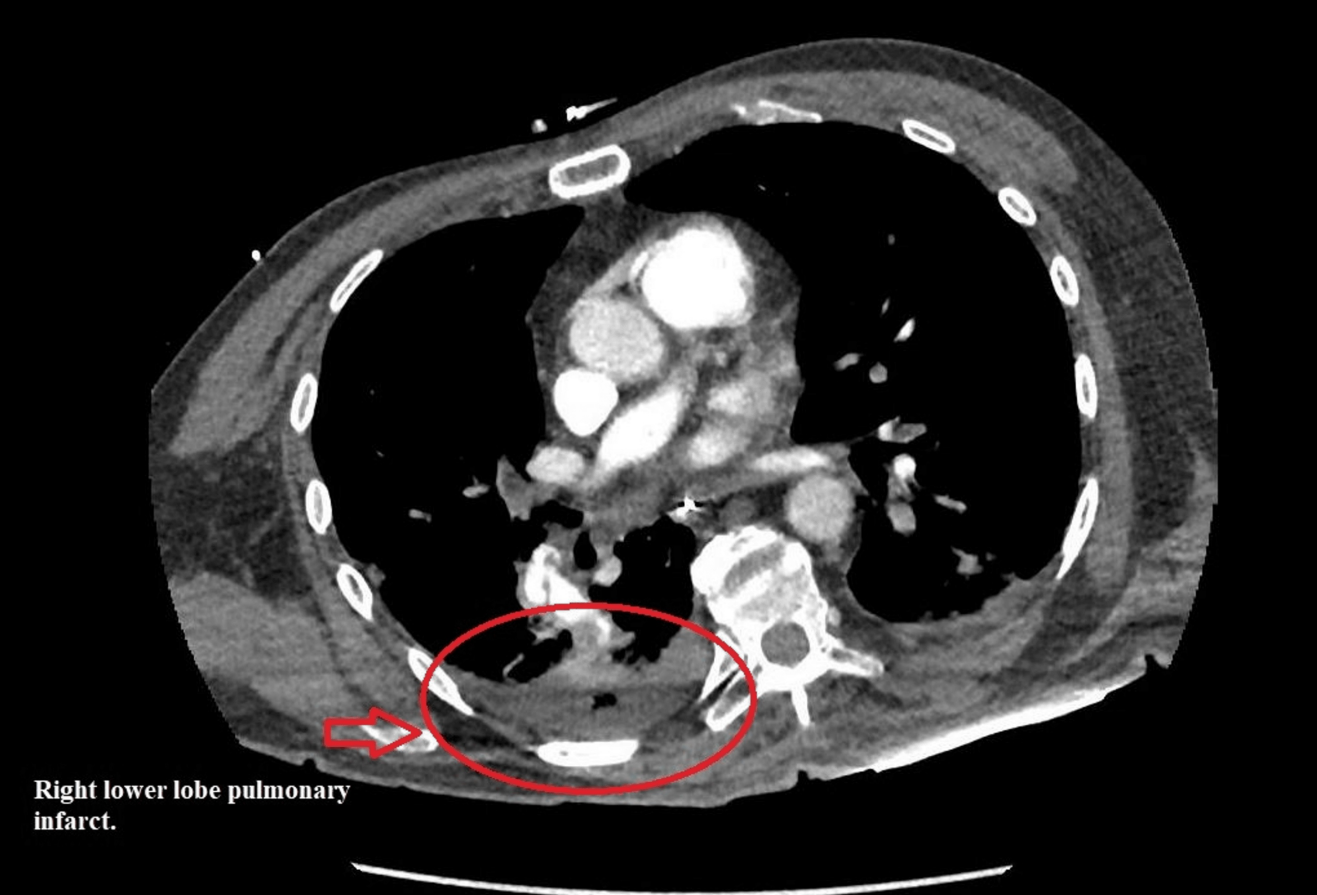
Computed tomography chest image demonstrating a peripheral wedge-shaped opacity in the right lung (arrow), representing pulmonary infarction secondary to distal pulmonary artery occlusion.

He subsequently suffered another PEA arrest, this time accompanied by an episode of ventricular tachycardia; one defibrillation shock was administered, and ROSC was achieved once more. Emergent chest computed tomography angiography (CTA) revealed an extensive burden of bilateral acute pulmonary emboli, with signs of right heart strain and multiple wedge-shaped infarctions in the lungs (Figures 4, 5).

Given his critical condition (persistent hemodynamic instability despite intubation and multiple vasopressors), the decision was made to administer systemic thrombolysis with a single bolus of tenecteplase. This intervention was initially complicated by transient bleeding from the oral cavity and hematuria, both of which gradually resolved with supportive care.

Over the next three days, the patient’s condition improved. He was weaned off vasopressor support and successfully extubated. At the family’s request, he was transferred to a tertiary care center for consideration of surgical pulmonary embolectomy or mechanical circulatory support. However, as he remained stable on therapeutic anticoagulation alone, no invasive intervention was deemed necessary. He was eventually discharged home in stable condition on long-term anticoagulation, with outpatient follow-up.

The patient and his family provided written informed consent for publication of this case and accompanying images. All identifying details have been omitted to protect patient confidentiality.

## Discussion

Massive PE is associated with high mortality, particularly when presenting with cardiac arrest [11]. In this setting, PEA is the most common presenting rhythm, reflecting acute right ventricular pressure overload leading to impaired left ventricular filling and circulatory collapse.

Echocardiographic findings of acute right ventricular dysfunction, including the classic McConnell’s sign (right ventricular free wall hypokinesis with apical sparing), are highly suggestive of PE in the appropriate clinical context [10]. A bedside cardiac ultrasound is especially valuable in unstable patients for whom transport to the radiology suite is unsafe. In this case, point-of-care echocardiography was instrumental in identifying right heart strain and raising suspicion for massive PE while other diagnostic measures were underway.

Systemic thrombolysis remains first-line therapy for massive PE in hemodynamically unstable patients, particularly when catheter-directed or surgical options are unavailable. Although thrombolytic therapy carries a risk of major hemorrhage, including an approximately 6.3% risk of major bleeding and a 2-5% risk of intracranial hemorrhage, it can be life-saving, with mortality reduction demonstrated in clinical registries and meta-analyses [4,12]. Our case highlights both the efficacy of thrombolysis and its bleeding risks, as the patient experienced only transient oral and urinary bleeding while ultimately achieving hemodynamic stabilization.

This case underscores the importance of maintaining a high index of suspicion for PE in patients presenting with recurrent PEA arrests, especially when risk factors for venous thromboembolism are present. It also illustrates the diagnostic utility of bedside echocardiography in identifying acute right ventricular strain and guiding urgent management decisions. Ultimately, it demonstrates that systemic thrombolysis, despite its associated bleeding risk, remains a vital treatment option in unstable PE when definitive reperfusion strategies (such as surgical thrombectomy or catheter-directed therapy) are not feasible or immediately available.

## Conclusions

Massive PE can present with recurrent cardiac arrest and requires a high index of suspicion for timely recognition. Bedside echocardiography, rapid initiation of anticoagulation, and consideration of systemic thrombolysis are central to the management of hemodynamically unstable patients. This case highlights the importance of integrating clinical findings, point-of-care imaging, and multidisciplinary decision-making and supports the potential benefit of prompt thrombolysis in life-threatening presentations.
